# How the Brain Retrieves Forgotten Memories

**DOI:** 10.1371/journal.pbio.0020283

**Published:** 2004-08-17

In the 2002 thriller *Memento*, the protagonist, Lenny, is plagued by a crippling neurological disorder that renders him incapable of storing memories for longer than fifteen minutes. He wants nothing more than to avenge the murder of his wife, but as another character in the film tells him, “Even if you get revenge, you're not going to remember it. You're not even going to know it happened.” Lenny has anterograde amnesia, a condition typically caused by stroke or other illness. (In the film, it's caused by a blow to the head.) Most forms of amnesia—including the more common retrograde amnesia, which involves the loss of long-term memory—are caused by some type of brain injury— particularly to the hippocampus.

Retrograde amnesia can arise from brain damage that interferes with memory storage, retrieval, or consolidation. And it is by studying retrograde amnesia that scientists have developed theories explaining where the brain stores memory traces and how it consolidates them into long-term memories. What ultimately causes amnesia—a failure to store memories or a failure to retrieve them—is not clear.

A major challenge in resolving this question experimentally is being able to determine whether an animal has truly recovered a lost memory or has simply re-learned the task at hand. By refining a classic behavioral neuroscience experiment to test spatial learning and memory, Livia de Hoz, Stephen Martin, and Richard Morris have developed a novel protocol that distinguishes retrieval from new learning. The experiment, developed by Morris over fifteen years ago, takes advantage of the fact that rats are good swimmers but prefer life out of water. Rats are placed in a pool and trained to remember the location of an escape platform, hidden just under the surface. (The rats can't see it because the water is cloudy.)

Here, de Hoz et al. trained rats to a particular platform location—their escape route—then gave them hippocampal lesions. One group received “sham” lesions, another partial lesions, and a third complete lesions. Partial lesions, the authors explain, should damage only a subset of the stored memory traces and thus weaken the rats' memory rather than completely disrupting it.

The rats' postoperative memory was tested by first placing them in the pool with the platform hidden: as expected, the lesioned rats couldn't remember where it was. After a minute, the platform was raised above the water, to remind them that it existed and that they could escape by climbing onto it. The platform was revealed in the original location for half the animals and in a novel location—which could be construed as misleading information if you're a rat—for the other half. If raising the platform was facilitating re-learning, then animals capable of learning would look for the platform in the new location. But if raising the platform functioned as a reminder, then animals should gravitate toward the place they were trained to, regardless of whether the platform reminder was in the original location or a new one. And that's what happened to the rats with partial lesions.

De Hoz et al. then repeated the experiments in a new environment. If the rats managed to escape in this situation, the authors explain, then the reminder treatment could be causing new learning rather than triggering recall of the original training experience. Not surprisingly, the rats with total lesions failed to learn in this new environment (or to be reminded in the previous experiment), while the control rats adapted to the new location. The group with partial lesions failed to learn.

Since partially lesioned rats responded to the reminder treatment by recalling the original platform location, their initial memory failure couldn't have resulted from a storage failure. And since they did not realize they could escape from the new location, the reminder didn't induce new learning. Among the many questions these results raise is what role the hippocampus plays in memory storage and retrieval. It could be, as the authors propose, that essential components of spatial memory traces are either stored in the hippocampus or reactivated there, since only the partially lesioned rats responded to reminders. Whether that proves true, de Hoz et al. have contributed a much needed resource for investigating the neural basis of memory loss.[Fig pbio-0020283-g001]

**Figure pbio-0020283-g001:**
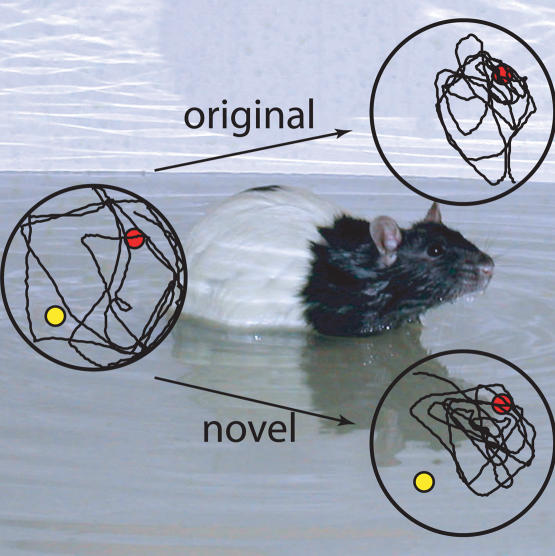
Rats given partial hippocampal lesions after training in a watermaze are initially unable to remember the location of the escape platform

